# Hidden in Plain Sight: Metastatic Renal Cell Carcinoma Masquerading as Multinodular Thyroid Disease

**DOI:** 10.7759/cureus.104340

**Published:** 2026-02-26

**Authors:** Catherine A Toal, Anna Aronova, Maryam Zenali

**Affiliations:** 1 Department of General Surgery, New York Medical College, Valhalla, USA; 2 Department of Surgery, Northern Westchester Hospital, Mount Kisco, USA; 3 Department of Pathology and Laboratory Medicine, Northwell Health, New Hyde Park, USA

**Keywords:** cytology, general surgery, histology, metastasis, metastatic renal cell carcinoma, multinodular thyroid, oncology, pathology, thyroid neoplasm

## Abstract

Metastases to the thyroid gland are uncommon, with renal cell carcinoma (RCC) representing a rare but clinically significant occurrence. RCC may metastasize after a prolonged dormancy and remain clinically silent for years before detection. We present a case of a 61-year-old man who initially presented with hematuria. Imaging at the time revealed RCC confined to the kidney. Several years later, during surveillance imaging for newly diagnosed prostate cancer via prostate-specific membrane antigen (PSMA) positron emission tomography (PET), bilateral thyroid nodules were incidentally detected. Histopathological examination of the resected multinodular thyroid revealed an unexpected finding of metastatic RCC, initially masquerading as a primary thyroid neoplasm. Awareness of RCC’s propensity to masquerade as a primary thyroid tumor is paramount for the surveillance and management of patients with a history of RCC.

## Introduction

Thyroid cancer is one of the five most common cancers in the United States, with papillary carcinoma comprising roughly 80-85% of all cases [1-3]. Thyroid neoplasms are often identified via aspiration cytology sampling of thyroid nodules and classified using the Bethesda System, with categories V (suspicious for malignancy) and VI (malignant) denoting the highest risk for malignancy [4]. Metastases to the thyroid are rare, occurring in fewer than 3% of patients undergoing surgery for thyroid neoplasms. As such, neoplastic cytology findings are often presumed to represent primary thyroid cancer (such as papillary carcinoma) rather than a secondary cancer [5].

When metastases to the thyroid occur, the most common primary sites include skin, lung, breast, and kidney [6,7]. Kidney cancers encompass a multitude of histologic and molecular subtypes, with clear cell renal cell carcinoma (RCC) forming the majority (~75%) of cases [8,9]. Due to the clinically silent nature of RCC, up to one-fifth of patients may have metastatic disease at the time of initial diagnosis [5,6,8]. Per reports, RCC accounts for up to one-third of secondary thyroid tumors yet represents only a small minority of all malignant intrathyroidal tumors [6,7]. As a result, metastatic RCC poses a significant diagnostic challenge as it can mimic primary thyroid neoplasms both clinically and radiographically.

We present a case of metastatic clear-cell RCC to the thyroid presenting as bilateral thyroid nodules, discovered incidentally on prostate-specific membrane antigen (PSMA) positron emission tomography (PET) performed for prostate cancer surveillance. The discovery of metastatic RCC occurred several years after initial RCC diagnosis and radical nephrectomy. This case highlights the importance of surveillance and consideration of metastatic disease in patients with a history of RCC, even when lesions are encountered several years after initial diagnosis and treatment.

## Case presentation

A 61-year-old man with a past medical history of basal cell skin cancer and a long-term occupational history at a nuclear power plant initially presented to the emergency room with hematuria and abdominal pain. A pelvic computed tomography (CT) scan revealed a 7 cm enhancing mass in the mid-to-lower pole of the right kidney, leading to a radical nephrectomy one month later. The kidney was excised with widely negative margins. Histopathologic examination revealed a 7.2 cm clear-cell RCC, Fuhrman nuclear grade 2 of 4, with a minor component of grade 3 [10]. Microscopic examination reaffirmed the negative status of the margins of resection. The tumor nodule was confined to the kidney, with pathologic staging pT2aNxR0 [11].

Seven years later, a PSMA PET scan, performed for staging of a newly diagnosed prostate cancer, incidentally highlighted bilateral thyroid nodules with paratracheal lymphadenopathy. No additional sites of metastatic disease were identified. The patient was not experiencing any thyroid-related symptoms at the time of the scan, and thyroid function tests were within normal range. A subsequent thyroid ultrasound reaffirmed the PET findings, showing a 3.8 cm right thyroid nodule (Thyroid Imaging Reporting and Data System {TIRADS} 4) and a 3.4 cm left thyroid nodule (TIRADS 3). Fine-needle aspiration of the right thyroid nodule was classified as Bethesda category V (suspicious for malignancy), and a total thyroidectomy ensued [4].

**Figure 1:**
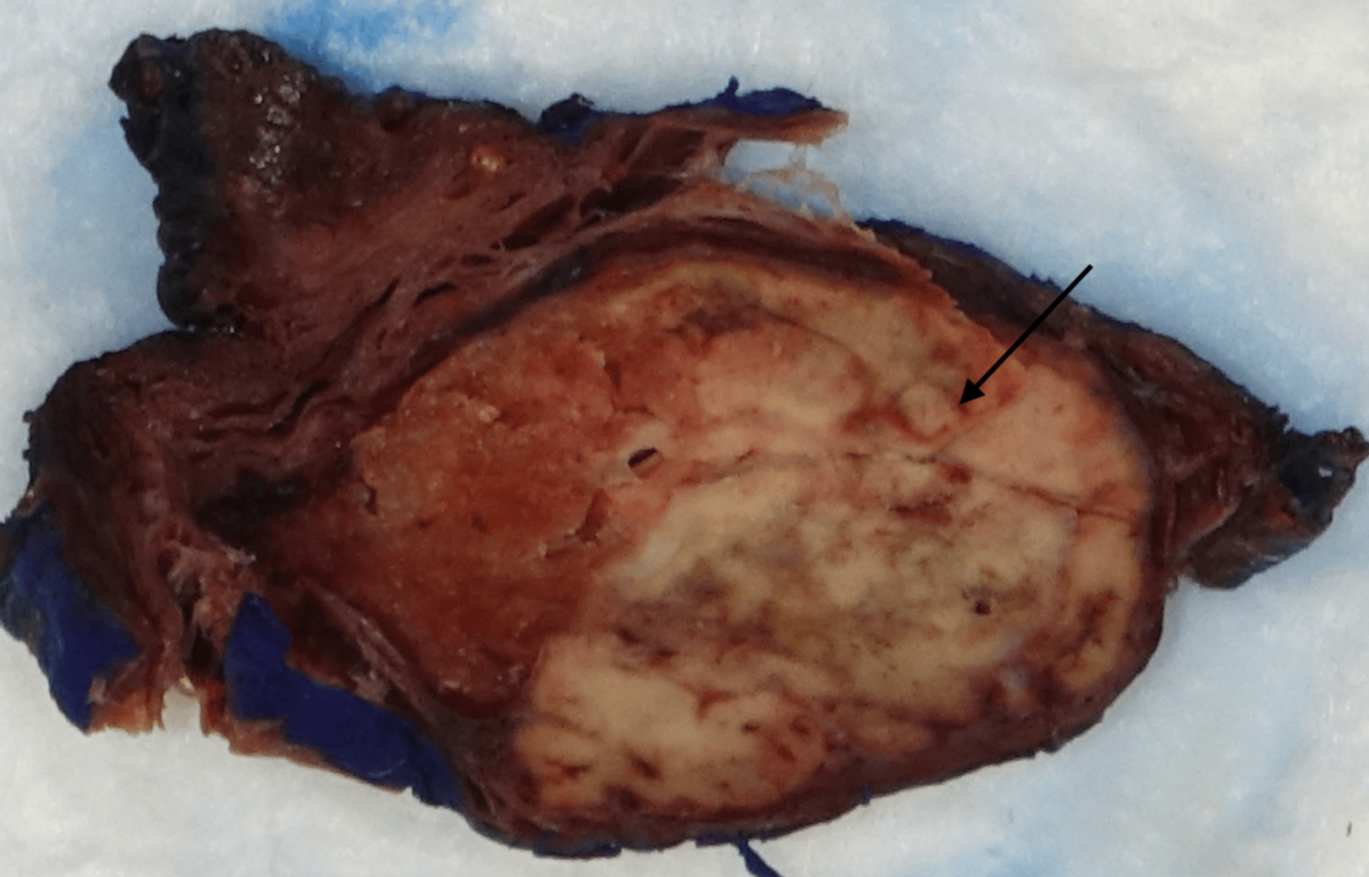
Macroscopic image of one of the metastatic nodules. A well-defined, variegated tumor mass with necrotic foci (arrow) is surrounded by a thin rim of thyroid tissue.

The resected thyroid gland consisted of a 9.5x5.6x4.0 cm right lobe and an 8.5x3.0x2.8 cm left lobe. On gross examination, the right lobe contained several nodules, including a prominent 5.5 cm yellow-tan necrotic and hemorrhagic mass; the left lobe was multinodular and contained a 1.8 cm tan, firm, and circumscribed mass (Figure 1).

**Figure 2:**
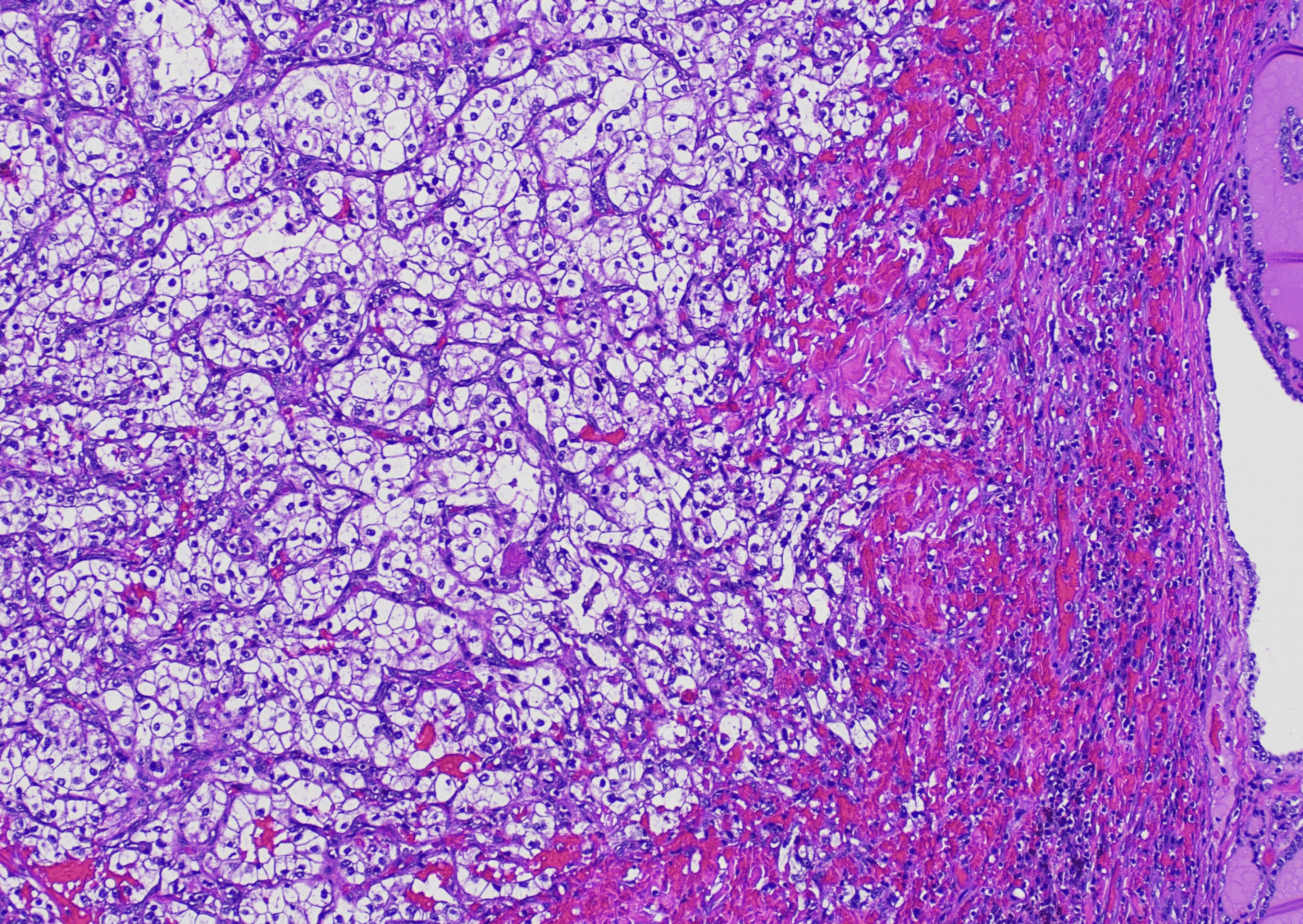
Optically clear to weakly eosinophilic polygonal tumor cells with mild to moderately irregular nuclei are arranged in nests and tubules (40x magnification).

**Figure 3:**
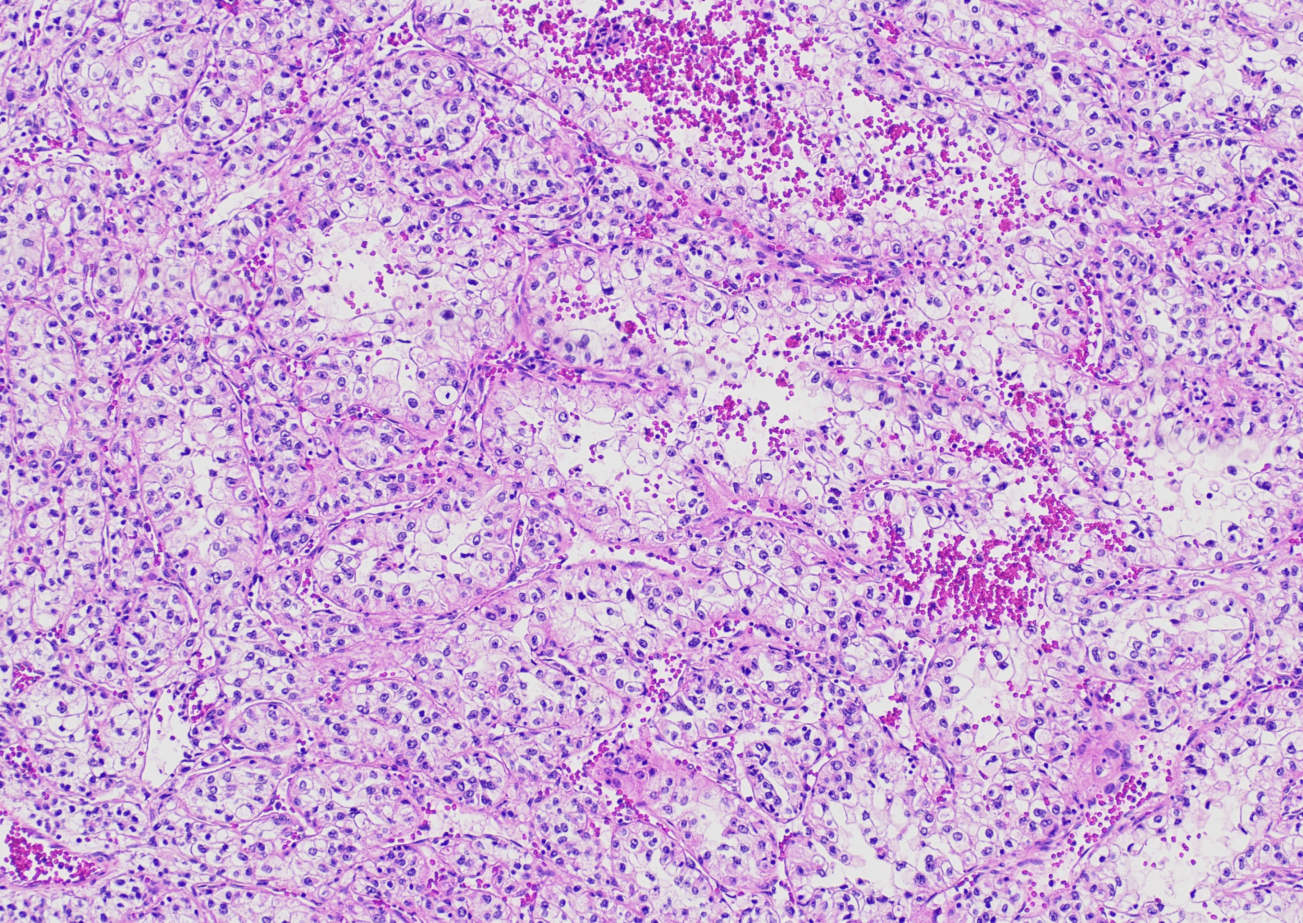
Aggregates of tumor cells with increased cellular pleomorphism and somewhat hobnail appearance are separated by an arborizing capillary network (100x magnification).

**Figure 4:**
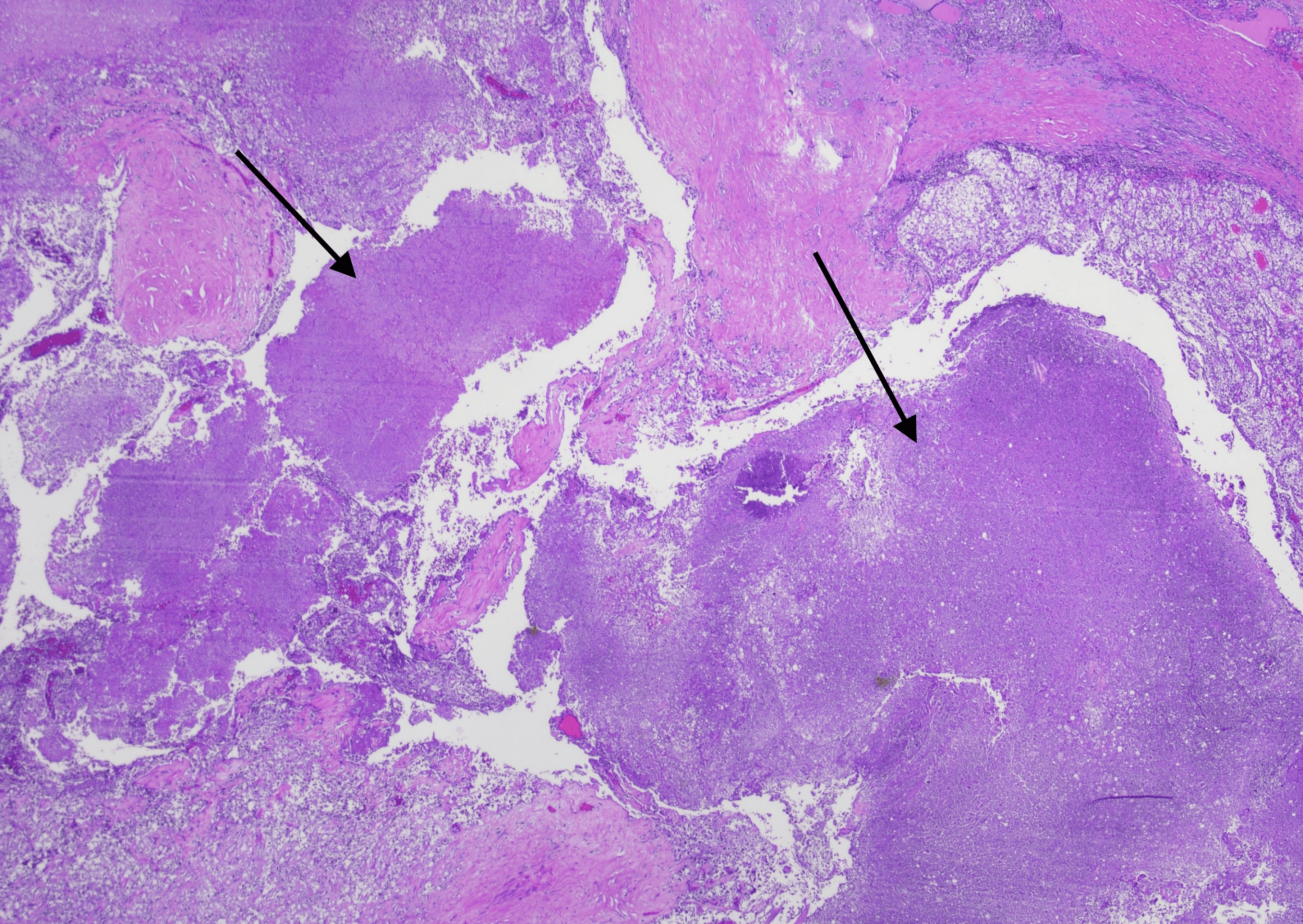
Foci of coagulative necrosis in metastatic tumor with a geographic configuration (arrows) (20x magnification).

On microscopic examination, the tumor cells had clear-to-eosinophilic cytoplasm and variably pleomorphic nuclei (Figures 2, 3). Additionally, there were lymphangitic tumor foci and geographic areas of necrosis (Figure 4).

**Figure 5:**
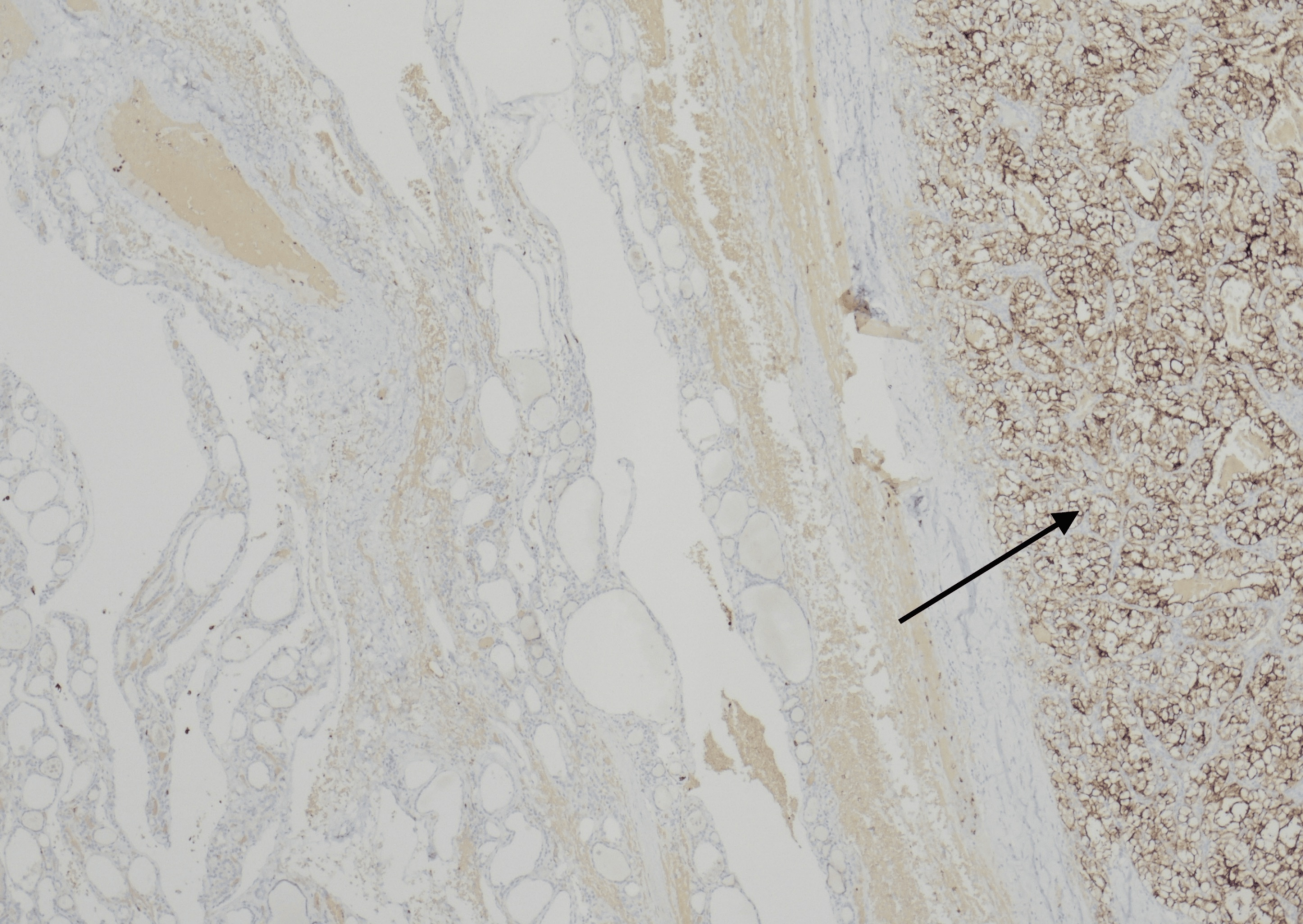
A photomicrograph of CD10 immunostain, where metastatic RCC (right aspect, indicated by arrow) is highlighted through differential staining (40x magnification). RCC has strong brown granular membranous staining, while the background thyroid is negative for specific staining. RCC: renal cell carcinoma; CD10: cluster of differentiation 10

**Figure 6:**
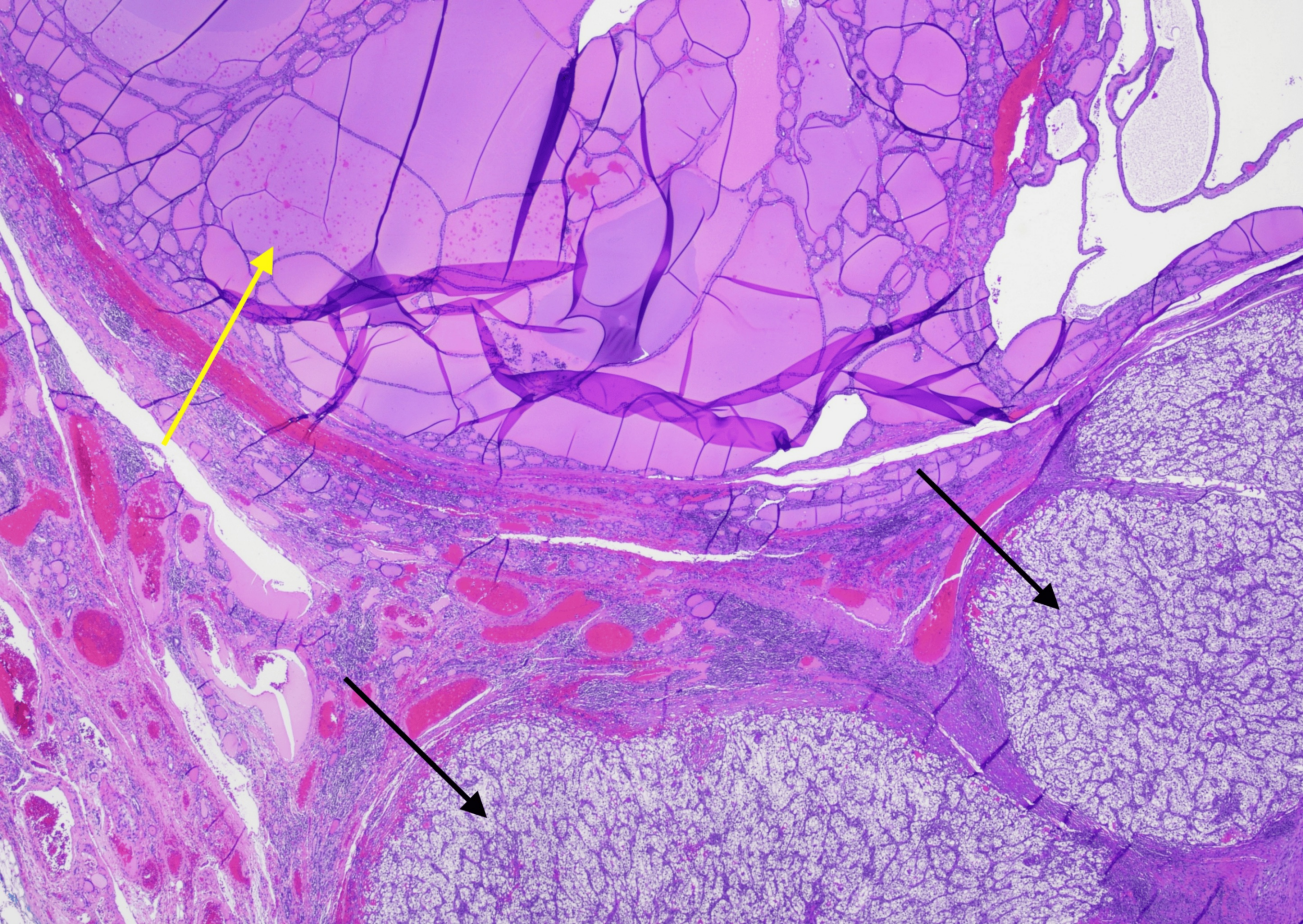
Nests of clear cell tumor nodules in lower aspect of the photomicrograph (indicated by black arrows) are surrounded by small aggregates of dense lymphoid tissue and nodular thyroid with variably-sized dilated follicles (indicated by a yellow arrow) (20x magnification).

The tumor was positive for paired box gene 8 (PAX-8), vimentin, epithelial membrane antigen (EMA), cytokeratin antibody cocktail 5.2 (CAM5.2), and cluster of differentiation 10 (CD10) (Figure 5). The tumor was negative for thyroid transcription factor 1 (TTF-1), cytokeratin 7 (CK7), cytokeratin 20 (CK20), and NK3 homeobox1 (NKX3.1) stains. In view of the tumor's morphology and immunohistochemical profile, a diagnosis of metastatic clear-cell RCC was rendered. Background thyroid demonstrated multinodular architecture with dense lymphoid aggregates and focal atypical B-cell infiltrate, consistent with small B-cell lymphoma on further immunophenotypic work-up (Figure 6).

Following thyroidectomy, the patient was started on levothyroxine thyroid hormone replacement therapy and remains alive and well two years after his surgery. The patient has been followed by the clinical urology service for RCC, with surveillance CT imaging every six months initially and then yearly imaging for three to five years. To date, there is no new evidence of metastatic RCC by imaging.

## Discussion

Metastases to the thyroid gland are uncommon, reported in 1.4-3% of patients undergoing surgery for thyroid neoplasms and in 1.25-4% of autopsy series [5]. Renal cell carcinoma accounts for 12-34% of secondary thyroid tumors, overall representing less than 0.1% of all thyroid neoplasms [6,7]. RCC is frequently detected late in the course of disease due to lack of symptoms, with up to 20% of patients presenting with metastases at the time of diagnosis, most commonly to the lung, bone, liver, pancreas, and brain [5-7,12]. Renal cell carcinoma has an unpredictable clinical course and metastasizes across a wide range of initial stages and grades. Even in patients with localized RCC treated with partial or total nephrectomy, approximately 30% of patients develop metastases later in life [8].

Diagnosis of metastatic RCC to the thyroid can be challenging, as it may mimic primary thyroid neoplasms clinically, radiographically, and occasionally cytologically. Further complicating detection, RCC may metastasize into a pre-existing thyroid neoplasm, a phenomenon termed tumor-to-tumor metastasis. Intriguingly, both RCC and thyroid neoplasms are frequent recipients of tumor-to-tumor metastasis. RCC is reported to be the most common donor neoplasm metastasizing to a primary thyroid disease; however, this phenomenon remains quite rare. Rich vascular supply of the thyroid gland and altered microenvironment in neoplastic tissue are among the proposed factors that may favor metastatic implantation. Conditions such as goiter, thyroiditis, or neoplasm may increase susceptibility due to the induction of metabolic changes or vascular insults. The mechanisms underlying tumor-to-tumor metastases are complex and remain poorly understood [7,13].

In cases of operable metastatic disease, management may include cytoreductive nephrectomy, oligometastasectomy, and systemic therapy (immunotherapy or targeted therapy) tailored to the tumor’s genomic profile, followed by active surveillance [8]. Active surveillance is crucial, but a consistent protocol has yet to be defined. One of the recommended surveillance protocols alternates between ultrasonography and CT or magnetic resonance imaging every three months in the first year, then extends to every six months in the second year and occurs annually thereafter [8]. Current treatments for metastatic disease include tyrosine kinase inhibitors and immune checkpoint inhibitors (ICIs), both of which have shown variable clinical responses in cases of metastatic RCC [14]. Cytotoxic T-cells in RCC seem to recognize tumor-specific epitopes, whose presence can be associated with responses to immune checkpoint blockade, but the effects are typically not durable [14,15]. Cell-based immunotherapy approaches such as chimeric antigen receptor T-cells (CAR T) and T-cell receptor-engineered T-cells hold promise. Cell-based immunotherapy has shown efficacy in hematopoietic cancers and is under clinical investigation for the treatment of RCC [14].

A definitive diagnosis of metastatic RCC requires histologic and immunohistochemical evaluation. Management of secondary thyroid tumors may involve surgery, chemoradiation therapy, and targeted cellular or immunotherapy [16]. Despite appropriate treatment, metastatic RCC remains associated with a high risk of mortality [8]. In a subset of patients with isolated metastasis to the thyroid, a total thyroidectomy has been proposed to optimize treatment and prevent further spread of the malignancy. Patients with secondary thyroid tumors who undergo surgical treatment have been observed to experience significantly improved overall survival compared with those receiving conservative management. The greatest survival benefit from metastatectomy has been observed in cases of metastatic kidney cancer to thyroid. In patients without widespread metastasis, survival rates are reportedly higher in patients with metastatic RCC to thyroid gland versus metastasis to non-thyroid tissues [6-8,12,16].

## Conclusions

Metastases of RCC to the thyroid gland are a rare cause of thyroid neoplasms. RCC can remain dormant and asymptomatic for several years after initial treatment, as in this case. Given RCC’s propensity to masquerade as a primary thyroid cancer clinically, radiologically, and cytologically, evaluation alone can be particularly challenging. This report illustrates a rare presentation of metastatic RCC presenting as new thyroid nodules, several years after initial treatment with right radical nephrectomy. This emphasizes the importance of maintaining a high index of suspicion for RCC metastases, even in patients with several years of disease-free survival. Additionally, physicians should consider tailored immunophenotypic examination in cases with atypical thyroid cytology or when clinical history raises a concern, as in this patient.

In patients with unique environmental exposures, such as occupational radiation exposure, multiple oncologic processes may coexist or arise through distinct pathologic mechanisms. Such patients may benefit from vigilant long-term follow-up care and a lower threshold for comprehensive diagnostic evaluation when new lesions are identified.
